# Benefits of Spectral Property Engineering in Distributed Brillouin Fiber Sensing

**DOI:** 10.3390/s21051881

**Published:** 2021-03-08

**Authors:** Cheng Feng, Thomas Schneider

**Affiliations:** THz Photonics Group, Institut für Hochfrequenztechnik, Technische Universität Braunschweig, Schleinitzstr. 22, 38106 Braunschweig, Germany; thomas.schneider@ihf.tu-bs.de

**Keywords:** stimulated Brillouin scattering, distributed fiber sensing, Brillouin optical time-domain analyzer, slope-assisted Brillouin optical time-domain analyzer, spectral property engineering

## Abstract

As one of the most consolidated distributed fiber sensors based on stimulated Brillouin scattering, the Brillouin optical time-domain analyzer (BOTDA) has been developed for decades. Despite the commercial availability and outstanding progresses which has been achieved, the intrinsic Lorentzian gain spectrum restricts the sensing performance from possible further enhancements and hence limits the field of validity of the technique. In this paper, the novel method of engineering the gain spectral properties of the Brillouin scattering and its application on static and dynamic BOTDA sensors will be reviewed. Such a spectral property engineering has not only provided improvements to BOTDA, but also might open a new way to enhance the performance of all kinds of distributed Brillouin fiber sensors.

## 1. Introduction

Besides the significant impact on telecommunications, the optical fiber, as arguably one of the most important inventions in the last century, plays also an important role in the field of sensing due to its easy embeddability, chemical inertia and immunity to electromagnetic interference. Different from the traditional point sensors, such as fiber Bragg gratings (FBG) [1], distributed fiber sensors employ the total fiber length as the sensing media, distributively interrogating the measurand change of every fiber section. Thus, a single fiber can act as thousands of point sensors and is favorable and will often be implemented for long range tasks, such as structural health monitoring and pipeline leakage surveillance [2].

Stimulated Brillouin scattering (SBS), as one of the dominant nonlinear effects in optical fibers, is a promising candidate for distributed fiber sensing owing to its unique characteristics of high gain and low threshold. The working principle of a distributed Brillouin fiber sensor can be classically described as the interaction between a forward propagating optical wave, called the pump, and a backward propagating frequency downshifted scattered optical wave, called the probe, mediated by an acoustic wave [3]. For the most consolidated time-domain distributed Brillouin sensing technique, also known as Brillouin optical time-domain analyzer (BOTDA), the pump wave is pulsed in the time domain to achieve the spatial resolved measurement, transferring energy to the probe wave according to the local Brillouin gain spectrum (BGS) [4]. Since temperature [5] and strain [6] at a distinct position of the fiber change the density at this fiber section, the propagation velocity of the acoustic wave will be altered. According to the energy and momentum conservation, the local temperature and strain can be determined by measuring the frequency difference between the pump wave and the peak of the local BGS, also known as the Brillouin frequency shift (BFS) [7]. Due to a limited signal level and the pulsed pump, however, the interaction length between the optical waves and thus the intensity change of the probe wave is quite low. Thus, a main drawback of a conventional static BOTDA configuration is the relative low signal-to-noise ratio (SNR). On the one hand, the limited signal level mainly results from the short interaction length required by the spatial resolution, and the power limits on the pump and probe wave due to the modulation instability (MI) [8] and non-local effects [9,10,11], respectively. On the other hand, the noise level of a conventional BOTDA system mainly comes from the laser source, (i.e., the relative intensity noise), and the photodetector, (i.e., the thermal noise and shot noise [12,13,14]), leaving little possibilities to improve. In the previous investigations, it is reported that, a more proper fitting algorithm could contribute to a more accurate determination of the BGS peak, i.e., higher measurand resolution [15,16]. Similarly, a sharp and better shaped BGS would also be more robust to the system noise and help to identify the BGS peak [17]. This can be easily achieved by engineering the spectral properties of the BGS.

In conventional static BOTDA, besides a time-consuming frequency scanning of the probe wave in the vicinity of the estimated BFS to reconstruct the BGS with an intrinsic Lorentzian shape [3], the limited SNR requires also a large number of averagings. Both of these disadvantages make the conventional BOTDA configuration ineligible for dynamic tasks, i.e., the sensing of fast changing environmental conditions. Thus, for dynamic sensing, there are several special designed BOTDA techniques. The fast-BOTDA [18] technique still employs frequency scanning but replaces the slow, synthesizer-based frequency switching by a programmed instantaneous frequency sequence. Despite the drastically reduced measurement time, dependent on the fiber length and the number of scanning frequencies, usually hundreds of microseconds are still required [18]. Multi-tone interrogation, also called sweep-free BOTDA [19,20,21,22], measures the full BGS by many pump-probe pairs at the expense of a quite complicated hardware and a few other constraints, such as the limited resolution of the acquired BGS and a signal distortion due to four wave mixing (FWM).

Though with some limitations, the fastest way of sensing is to totally skip the frequency scanning process. Slope-assisted BOTDA (SA-BOTDA) [23,24] takes the advantage of the linear spectral response between the frequency shift and the Brillouin gain on the (rising and/or falling) edge of the BGS. The measurand variations shift the BGS in the frequency domain, resulting in corresponding changes of the Brillouin gain at the interrogation frequency. The interrogation point of an SA-BOTDA was set at the 3 dB point of the BGS at its first proposal [24] and the measurement was not truly distributed. Later on, the interrogation point is moved to the inflection point of the BGS for a better sensing performance [25]. Further improvements on the technique have enabled not only a truly distributed measurement [23], but also a pump-power independent measurement of strain and/or temperature [26]. There are two main sensing metrics of this techniques, namely the slope and the linear range [27]. The former one symbolizes the smallest detectable measurand change and is defined as the conversion factor between a BFS shift and the corresponding gain change. The latter one is characterized by the span of the frequency range with the quasi-linear spectral response. Despite the various quantitative criteria [28,29,30], they all symbolize the largest measurand change that the technique could measure. However, the main drawback of this consolidated technique lies in the trade-off between the linear range and slope, which prevents them from any simultaneous enhancements [31]. Since long, the community has tried large efforts to widen the dynamic range with various novel methods [28,29,30], all at a cost of flattened slope. This could be further attributed to the intrinsic limitation of the spectral property of the BGS.

Based on the above analysis, despite the well-development of the present BOTDA techniques, the engineering of the gain spectrum could further enhance not only the performances of static sensing but also that of the demanding dynamic sensing. The focus of this paper is to review the specific benefits of gain spectrum engineering for sensing. The content of this paper is three-folded. It begins with the general description and the principle of the gain spectrum engineering in Section 2. Section 3 and Section 4 investigate the application of gain spectrum engineering in the conventional static BOTDA and dynamic SA-BOTDA techniques and their performance enhancements, respectively.

## 2. Engineering of the Spectral Properties of Brillouin Scattering

Since the shape of the Brillouin gain is the convolution between the pump spectrum and the material dependent intrinsic Lorentzian gain spectrum of the SBS, a broadening of the SBS gain is relatively easy. Thus, there are several ways to engineer the gain spectral property of SBS by a direct [32,33] or external [34,35] modulation of the pump wave. The principle of pump modulation for gain spectrum engineering can be mathematically expressed as: [36]

(1) $$G_{SBS}^{eff}\left(\nu \right)=G_{SBS}\left(\nu \right)*\frac{P_{p}\left(\nu \right)}{P_{p}^{tot}}$$

where ∗ denotes the convolution operator, $P_{p}\left(\nu \right)$ is the pump spectral density, $P_{p}^{tot}$ is the total pump power given by the integral of the spectral density over the full frequency range as $P_{p}^{tot}=\int_{0}^{\infty }P_{p}\left(\nu \right)d\nu$, $G_{SBS}$ is the intrinsic Lorentzian BGS of a monochromatic wave and $G_{SBS}^{eff}\left(\nu \right)$ is the overall SBS spectral response of the polychromatic pump wave. The method provides the possibility to achieve any arbitrary, high quality BGS profile when nonlinear and experimental insufficiencies are compensated by a feedback loop and controlling software [37,38]. However, due to the mathematical characteristic of the convolution operation, the overall SBS spectral response cannot be narrower than the intrinsic Brillouin linewidth and this intrinsic linewidth is defined by the lifetime of the phonons in the material [39,40]. However, especially for sensor applications a sharper, steeper and deeper gain would be preferable [7]. Although the shape of the Brillouin gain is a material dependent function, there are three methods to decrease the SBS gain bandwidth, i.e., the gain enhancement in a multistage SBS process [41], the frequency domain aperture [42] and the superposition of gain and loss(es) [43].

The linewidth of the SBS can be reduced by increasing the gain. However, for high gains the probe wave gets saturated and a further enhancement is not possible in a single stage SBS system. If a multistage system is used instead, the probe can be attenuated before it is injected into the next stage [44]. Thus, with every stage the gain gets higher and the linewidth narrower [41]. For a loop system like a Brillouin laser, the gain can become extremely narrowband. However, up to now this method has not been used for sensing.

The frequency domain aperture method takes advantage of the inhomogeneous Brillouin gain saturation [45]. It can be described as the dependence of the Brillouin amplification on the probe wave signal, when the probe power is comparable to the pump power. The inhomogeneous gain saturation characteristic enables the saturation effect to occur only at the specific probe frequency (gain suppression) without interferences to the gain processes in other frequencies of the BGS. In this way, the Brillouin linewidth can be drastically narrowed to only 3 MHz [42]. However, for sensing this method has not been incorporated so far.

**Figure 1:**
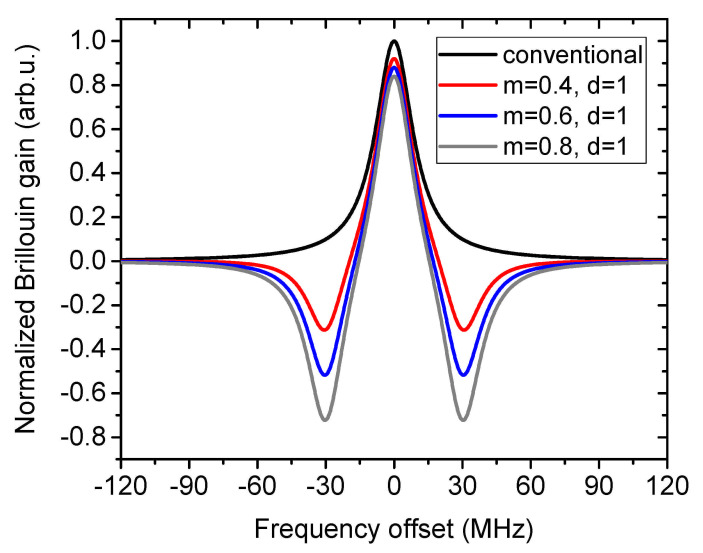
Engineered BGS by the superposition of a gain with two symmetric losses.

More degrees of freedom for BGS engineering are provided by the superposition method, which makes it more favorable for sensing applications and the focus of this paper. It can be described as the conventional Brillouin gain superimposed by two symmetric Brillouin losses with a frequency offset [43]. The engineered BGS can be well modeled by: [43,46]

(2) $$G_{eng}\left(\nu \right)=G_{SBS}\left(\nu \right)-m\cdot G_{SBS}\left(\nu +d\cdot \gamma_{B}\right)-m\cdot G_{SBS}\left(\nu -d\cdot \gamma_{B}\right)$$

where $G_{SBS}\left(\nu \right)$ is the conventional BGS specifically written as:

(3) $$G_{SBS}\left(\nu \right)=\frac{\frac{1}{2}g_{0}P_{p}L_{eff}}{1+4{\left(\nu -\nu_{B}\right)}^{2}/{\gamma_{B}}^{2}}$$

with $g_{0}$ as the center SBS gain coefficient, the full-width at half maximum (FWHM) of the intrinsic Brillouin linewidth as $\gamma_{B}$, $P_{p}$ as the pump power and $\nu_{B}$ as the BFS, ν as the frequency offset between pump and probe wave. $L_{eff}=\left[1-\exp(-\alpha L)\right]/\alpha$ is the effective fiber length with α=0.2 dB/km as the attenuation coefficient and *L* as the physical fiber length. *m* is the ratio of the maximum Brillouin loss and gain, $d=\Delta /\gamma_{B}$, where Δ is the frequency offset between the center of the Brillouin loss and gain. In Figure 1, some typical superimposed gain spectra with different *m* and *d* values are depicted together with the conventional BGS with 30 MHz FWHM linewidth. In comparison with the conventional BGS, the superimposed BGS with narrowed linewidth is sharper in the vicinity of the peak gain, leading to an easier determination of the peak position in a noise environment. This is important for Brillouin based microwave photonics [47,48,49], spectroscopy [50,51,52], quasi-light storage [53] and distributed sensing [46], which will be reviewed in the next section.

## 3. Benefits of Brillouin Spectral Property Engineering in Static Distributed Sensing

**Figure 2:**
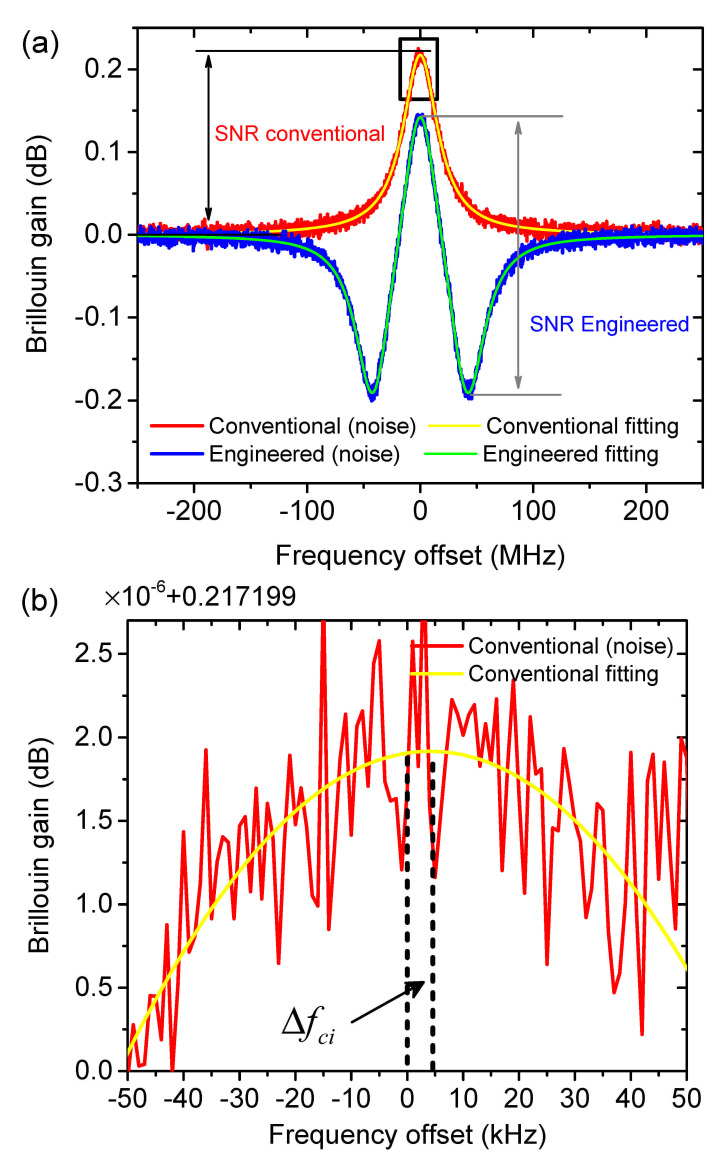
(**a**) Typical conventional (red) and engineered (blue) simulated BGS (d=1) with the same noise level and their corresponding fitting curves. The conventional BGS is excited by a 100 ns pump pulse with a spectrum FWHM of 54 MHz; (**b**) the magnified peak area of the conventional BGS (rectangular area in (**a**)), highlighting the BFS estimation error $\Delta f_{ci}$ due to the noise in the *i*th measurement.

For the overall BOTDA sensing performance especially an SNR enhancement would be of significant importance. A high SNR could extend the sensing range, avoiding the signal being ambiguous to the noise at the fiber end. With a high SNR the number of required averaging could also be reduced, leading to a drastically speed up of the measurement. More importantly, a higher SNR might contribute to a more accurate determination of the local BFS at a distinct fiber section, i.e., a higher measurand resolution. The traditional de-noise [54,55,56,57] and signal amplification [58] methods, however, cannot effectively improve the SNR of a conventional BOTDA setup. This can be mainly attributed to the consideration of Brillouin gain interaction only. If the superposition of the Brillouin loss(es) is considered, the SNR can be improved, as shown in Figure 2a. For a standard BOTDA system the SNR is the intensity difference between the probe or signal in the maximum of the Brillouin gain and far away from this point, as depicted in red. For the superposition with two losses, the overall Brillouin gain will be reduced. However, provided that the system noise level remains unchanged, the SNR for the engineered SBS interaction is the intensity difference between the maximum of the gain and the maximum of the loss, as shown in green.

### 3.1. Simulation

To verify the above mentioned analyzed benefits from the gain spectrum engineering, a simulation is carried out for quantitative prediction. Since the engineered BGS has a totally different shape as the Lorentzian or Voigt function, the conventional analytical way for error evaluation [59] is not valid. Instead, the original statistical way for error evaluation is used, which is composed of the following steps.

The conventional and engineered BGS at the pump launching end of the fiber are simulated by Equations (2) and (3), respectively, with a pump peak power of $P_{p}=20$ dBm to avoid MI [8] and a pulse width of 100 ns to calculate the effective fiber length $L_{eff}$. As discussed in Section 1, the noise of a conventional BOTDA system comes from the detector and laser source [12,13,60], which all have a uniform distribution in the frequency domain. Therefore, the noise performance is simulated by additive white Gaussian noise (AWGN). If we assume the probe power to be −14 dBm to avoid non-local effects [9], in comparison to the thermal noise from the detector, the shot noise and relative intensity noise are negligible [12,60]. Thus, the noise level on the conventional and engineered BGS should be the same, as in Figure 2a. Just for simplification, the conventional and engineered BGS (without noise) are originally centered at a zero frequency offset (to the BFS). Voigt function fittings [61] are applied to the conventional and engineered BGS with noise to identify the peak frequency. Due to the influence of noise, the identified peak frequency (estimated BFS) may not always be at zero. The offset between the estimated BFS and the precise BFS (at zero frequency) in the *i*th measurement is denoted as $\Delta f_{ci}$ and $\Delta f_{pi}$ for the conventional BGS and the proposed engineered BGS, respectively, as demonstrated by the single fitting for the conventional BGS in Figure 2b. To characterize the accuracy of the BFS estimation statistically, the standard deviation of the estimated BFS is calculated after a large number of measurements. In this simulation, the number of measurements *N* is set to 500. The advantage of the proposed engineered BGS is quantitatively symbolized by the ratio of the two standard deviations as: [46,62]

(4) $$\xi =\sqrt{\sum_{i=1}^{N}\Delta f_{pi}^{2}/\sum_{i=1}^{N}\Delta f_{ci}^{2}}$$

**Figure 3:**
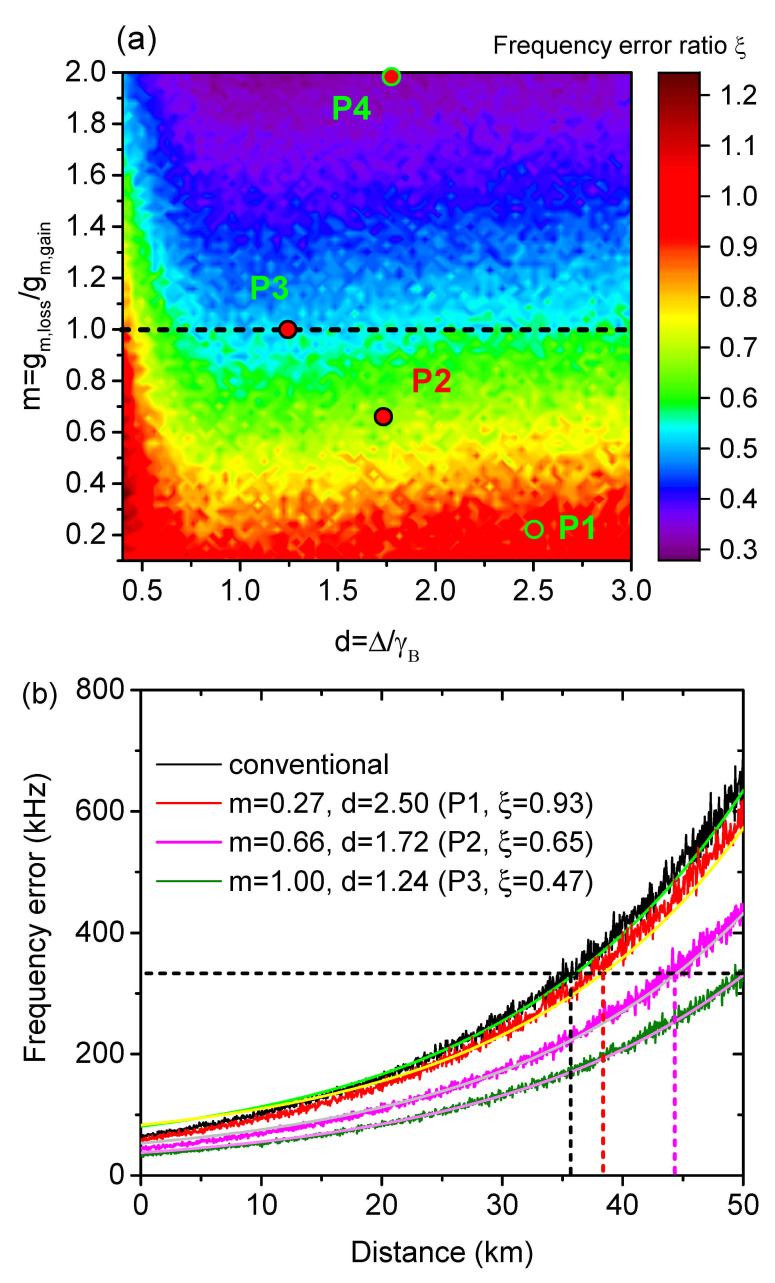
Simulation results of: (**a**) the frequency error ratio ξ as a function of *m* and *d*; (**b**) the distribution of the BFS determination error with the conventional and engineered BGS with selected *m* and *d* values in (**a**) marked as P1–P3.

The simulation result ξ is demonstrated as a function of *m* and *d* in Figure 3a. It is clearly indicated from Equation (4) that, the proposed engineered BGS is advantageous when ξ<1, which is the case in most of the situations in Figure 3a. The best performance is achieved with the lowest ξ value of 0.282 for m=2 and d=1.78, highlighted as P4 in Figure 3a. However, m>1 is impractical, which will be described in detail in Section 3.2. Therefore, the best practically achievable performance is located at P3 (m=1 and d=1.24) with a doubled measurand resolution (ξ=0.47). If the pump power suffers only from the intrinsic fiber loss, the frequency error distribution could be calculated in the same way with an extra factor of exp(−2αz) in Equations (2) and (3), where *z* is the distance to the pump launching end. As shown by the calculated results in Figure 3b, the (dis)advantage of the frequency error at the fiber start is well maintained until the fiber end. Under the same BFS error tolerance of 330 kHz (horizontal dashed line), the sensing range of the conventional BGS and the engineered BGS at P1, P2 and P3 are measured to be 35.6 km, 38.3 km, 44.4 km and 50 km, respectively, indicating that a sensing range extension of up to 40.4% has been achieved for the best case. The BFS error reduction and the corresponding sensing range extension can be physically interpreted as a higher signal level within a narrow frequency range, i.e., an overall sharper spectrum, which is more robust to the noise under the same level of peak determination. Please note that, due to the sufficient SNR for a signal detection, the sensing range here is still the physical length of the fiber, i.e., the whole fiber is under interrogation. The sensing range extension is quantified under the same frequency error tolerance, which requires not only a successful signal detection, but sets a further demand on the measurement error.

### 3.2. Implementation

Generally, there are three ways to implement the engineered BGS experimentally, namely the post-superposition, the multi-pump wave and the multi-probe wave scheme.

#### 3.2.1. Post-Superposition Scheme

The easiest way to achieve the proposed engineered BGS is to literally sum up the separately acquired conventional BGS and two Brillouin loss spectra from a conventional BOTDA setup. In this way, the multi-probe waves are not simultaneously launched but sequentially. The simplicity of the setup is the biggest advantage of this post-superposition scheme. By changing the pump power and RF modulation frequency on the probe wave, the *m* and *d* values can also be arbitrarily tuned.

However, the main disadvantage of this scheme is that a three times higher measurement time as compared to the other two variants is needed. Another disadvantage lies in the non-equivalence of the post-superimposed gain spectrum to the engineered BGS in the aspect of noise. Practically, not only the gain spectra but also their noise content are post-superimposed. Provided that the noise in the detector is mainly thermal and shot noise, the noise level on the gain probe wave is the same as on the loss probe waves, i.e., $\sigma_{g}=\sigma_{l1}=\sigma_{l2}=\sigma_{conv}$, where $\sigma_{g}$, $\sigma_{l1}$, $\sigma_{l2}$ represents the noise level of the gain probe wave and the two loss probe waves, and $\sigma_{conv}$ is the noise level of the probe wave for a measurement of the conventional BGS in the same time. The post-superposition of the probe powers including the noises leads to:

(5) $$\sigma_{eng}^{2}=\sigma_{g}^{2}+\sigma_{l1}^{2}+\sigma_{l2}^{2}=3\sigma_{conv}^{2}\;⟹\;\sigma_{eng}=\sqrt{3}\sigma_{conv}$$

Thus, this method has a $\sqrt{3}$ times higher noise level than the conventional one. Considering the sequential launched probe waves have extend the measurement time by a factor of 3, for a fair comparison, the measurement time with the conventional BGS must also be extended by 3 leading to a noise level reduction from the original $\sigma_{conv}$ to $\sigma_{conv}/\sqrt{3}$. Therefore, the noise level of the post-superimposed engineered BGS is 3 times higher than the conventional BGS, which results in a $\sqrt{3}$ times higher frequency error [59]. Considering the best advantage on the frequency error predicted by the simulation and the penalty by the post-superposition, the best measurand resolution is 47%$\times \sqrt{3}\approx$ 81.4% to the conventional BGS. Despite the remaining advantage, it is not the optimal option.

#### 3.2.2. Multi-Pump Wave Scheme

**Figure 4:**
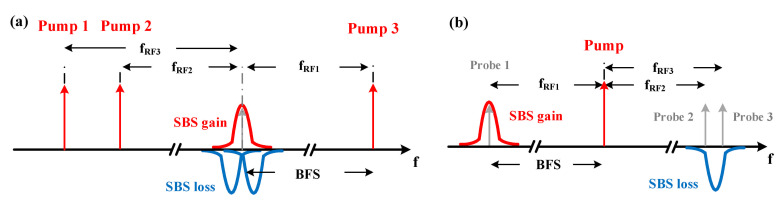
Schematic of (**a**) multi-pump wave and (**b**) multi-probe wave scheme. Red arrows mark the pump and gray arrows the probe waves.

An engineered BGS can be achieved by the Brillouin interaction between three simultaneous pump pulses and one continuous probe wave. An SBS pump wave generates a gain for a frequency down-shifted and a loss for an upshifted probe wave. Thus, as depicted in Figure 4a, pump 3 transfers energy to the probe wave, performing Brillouin gain interaction, while pump 1 and 2 take energy from the probe wave, producing Brillouin losses. According to Equation (2), the required relative frequencies between the pump and probe waves can be written as:

(6) $$f_{RF1}=f_{RF2}+d\cdot \gamma_{B}=f_{RF3}-d\cdot \gamma_{B}$$

where for the sake of frequency stability, the pump and probe waves are generated from the same laser source with radio frequency (RF) modulations. The BGS reconstruction can be carried out by scanning $f_{RF1}$ in the vicinity of the BFS. Meanwhile, $f_{RF2}$ and $f_{RF3}$ are also scanned correspondingly.

The biggest advantage of the multi-pump wave scheme is the full flexibility to choose *m* and *d*, i.e., *m* can be tuned by the corresponding pump pulse powers, which can be separately amplified and attenuated in the experimental condition, while *d* can be changed by the RF frequencies according to Equation (6). Due to MI, however, values of m>1 are not beneficial. The reason can be explained as follows: the MI determines the same maximum pump power for the gain and loss pulses even in the absence of the probe wave [8]. Therefore, the only way to achieve m>1 is to reduce the gain pump power. However, this reduces the SNR of the overall spectrum and reduces the sensor performance.

The disadvantage of the multi-pump scheme lies in the SNR penalty due to FWM. Owing to the co-propagation of high pump power pulses, the pump energy will be transferred to the idler frequencies, leading to a drastically reduced SNR [63]. In order to avoid this, a manual time delay, for instance, via a fiber Bragg grating [64] can be introduced. However, this complicates the setup and brings also other constrains.

#### 3.2.3. Multi-Probe Wave Scheme

Another option to implement the proposed engineered BGS is an interaction between three simultaneous probe waves and one pump pulse. As illustrated in Figure 4b, probe 1 is located in the BGS of the pump pulse, while probe 2 and 3 are within its Brillouin loss spectrum with an offset between each other. Since the probe waves are generated separately, the experimental setup would share the same complexity with the multi-pump wave scheme. The engineered BGS is reconstructed by summing up all three probe wave powers directly at the detector. The same scanning mechanism can be utilized according to Equation (6). For a negligible pump depletion, the Brillouin gain and loss are approximately the same in this scheme, namely m=1. However, since the best performance is predicted to be for m=1, this is no disadvantage. Besides the fact that in this scheme *m* is fixed to one, the reconstructed BGS is totally equivalent to that of the multi-pump wave scheme. However, due to the low power of the probe waves, the FWM between them is negligible. Therefore, in comparison to the other methods, the multi-probe wave scheme is the best option.

### 3.3. Results

**Figure 5:**
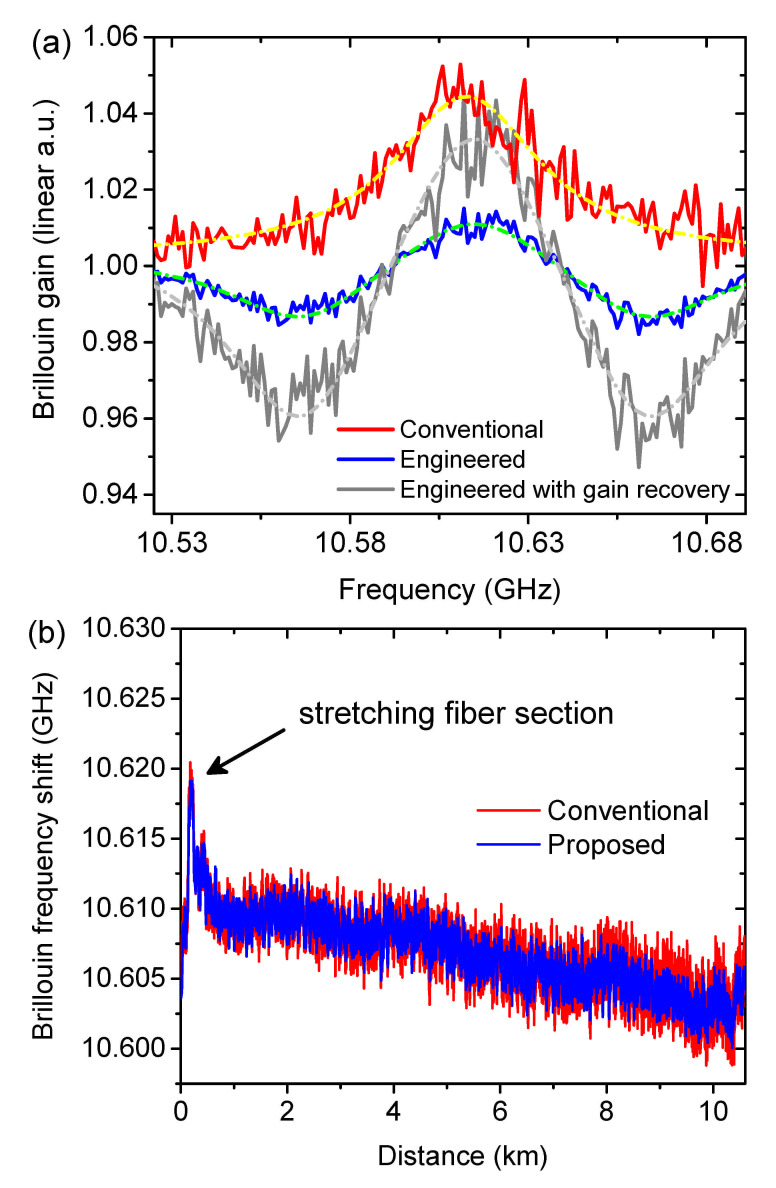
Experimental results of: (**a**) the reconstructed conventional (red) and engineered BGS with (gray) and without (blue) gain recovery; (**b**) the estimated BFS distribution with a stretching section.

Based on the above mentioned analysis, the multi-probe wave scheme with the settings of P3 (m=1 and d=1.24) is employed for experimental validation to achieve the best performance. To have a direct comparison, the pulse width, pump and probe wave powers are set to be exactly the same as in the simulation. A sufficient high extinction ratio of the pump pulse is ensured to avoid non-local effects [65,66,67]. A 10.6 km fiber is used as the sensing medium. The acquired conventional and engineered BGS at 10 m is depicted in Figure 5a with their corresponding Voigt fitting curves. The FWHM of the conventional BGS (red) is measured to be 54 MHz, showing good agreement with the assumption in the simulation. However, the amplitude of the acquired engineered BGS (blue) in Figure 5a is not showing consistence with the simulation. The reason for that is the division of the sensing response (trace) by the summation of all three probe powers (trace baseline) to calculate the Brillouin gain. Under small gain approximation, this gain penalty is a factor of 3. A detailed explanation, calculation and justification will be given in in Section 5.

**Figure 6:**
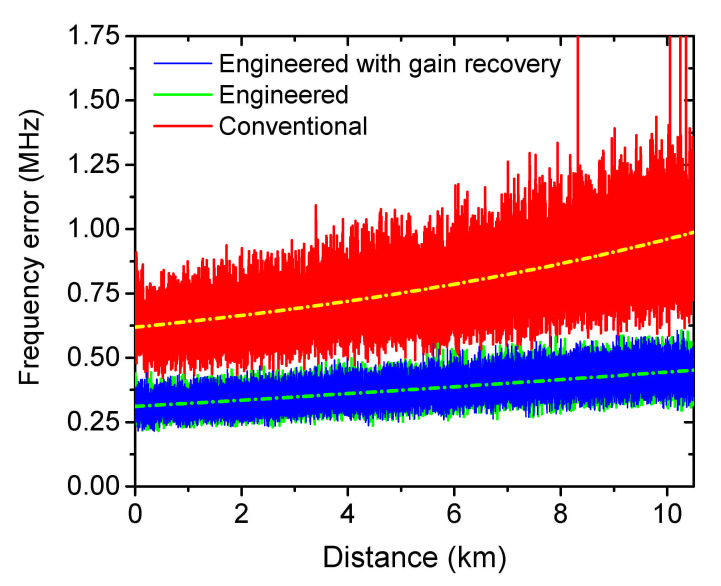
The frequency error distribution measured by the conventional (red) and engineered BGS with (green) and without (blue) gain recovery.

The estimated BFS from the Voigt function fitting is depicted in Figure 5b. It is clearly indicated that, the BFS distributions from both spectra are well overlapped and the one from the engineered BGS shows an even clearer trace, indicating a better functionality in accurate BFS estimation. In order to verify this, 48 measurements have been carried out consecutively. The BFS error distribution is calculated by the root-mean-square (RMS) value of the acquired 48 BFS values from the fitting curves at the same fiber section. As depicted in Figure 6, the frequency error from the engineered BGS is only 45.7% of that from the conventional BGS (quantified according to the dash dotted curves at the end of the fiber), showing very good agreement with the simulated predictions. Thus, under the same frequency error tolerance, the sensing range of the BOTDA system can be drastically extended by SBS spectrum engineering.

## 4. Benefits of Brillouin Spectral Property Engineering in Dynamic Distributed Sensing

Most of the dynamic sensing tasks aim to measure the strain or vibrational signals on the sensing fiber, such as structural health monitoring of skyscrapers, the turbulence on airplane wings and offshore wind turbines, for instance. Compared to static sensing, dynamic sensing has major differences in the performance requirements. This include: (a) a short measurement time in comparison to the measurand variation period and (b) a short sensing fiber length, usually in the range of several 100 m, which enables high pump and probe powers, due to weaker nonlinear effects. The latter one reduces the requirement on the number of averaging drastically and thus contributes to a shortened measurement time. Among all dynamic sensing methods, the slope assisted (SA)-BOTDA is arguably the most practical dynamic distributed Brillouin sensing technique up to now. However, the intrinsic spectral property of the Brillouin gain sets a trade-off between the maximum and minimum detectable measurand. In this section, gain spectrum engineering will be applied to provide a solution, so that the dynamic range (linear range) can be extended without compromise to the slope [27].

### 4.1. Theory

**Figure 7:**
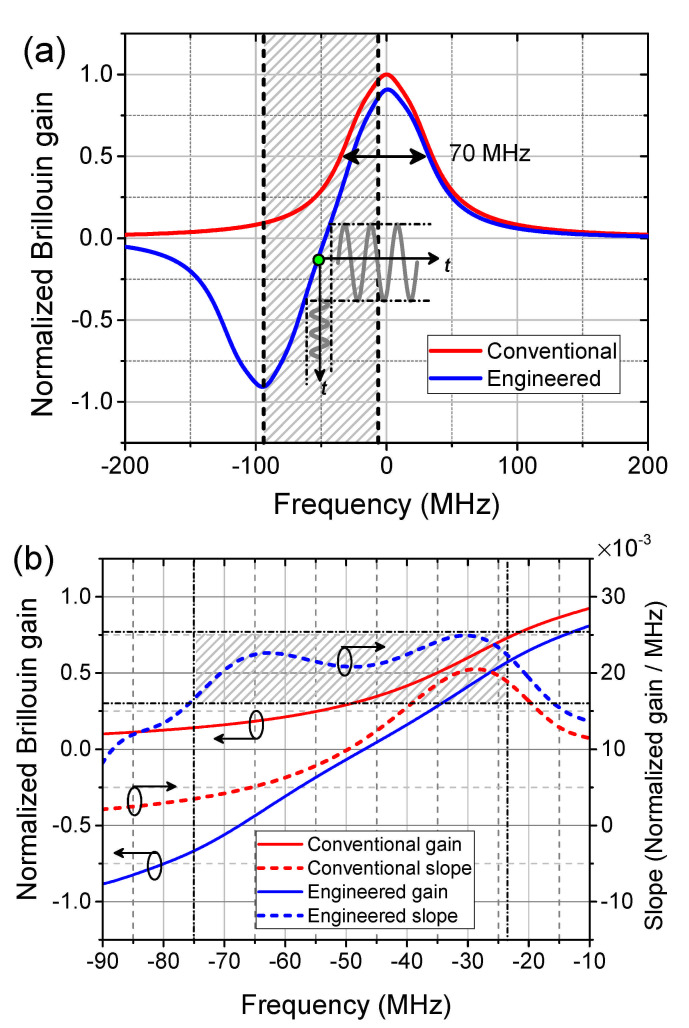
Simulation results of: (**a**) the conventional (red) and engineered (blue) BGS with the schematic principle of SA-BOTDA at an arbitrary interrogation point (green dot); (**b**) the slope of the conventional (red dashed) and engineered (blue dashed) BGS in the shadowed frequency area in (**a**).

Mathematically the engineered BGS for the dynamic sensing can be expressed as:

(7) $$g_{s}\left(\nu \right)=V\left(\nu \right)-m\cdot V\left(\nu +d\cdot \gamma_{B}\right)$$

where V(ν) is the Voigt function that describes the BGS when the pump pulse width is short [61], and *m* and *d* have the same definitions as in Equation (2). Different from the spectrum engineering in Section 3, only a single loss spectrum is superimposed at the edge of the gain. This will bring benefits to avoid the interference between two loss probe waves and a wider extension of the dynamic range. In Figure 7a, the conventional BGS (red) is simulated from the Voigt fitting curve of an experimentally achieved conventional BGS curve excited by a 14 ns pulse with an FWHM broadened to 70 MHz. The engineered BGS (blue) is constructed with *m* = 0.845 and *d* = 1.337 in Equation (7). An obviously extended linear range is built between the maximum gain and maximum loss in the frequency domain. The first derivative of the BGS (slope) in the shadowed frequency area in Figure 7a is calculated and depicted in Figure 7b. Compared to the conventional BGS, the flattened blue dashed curve in the shadowed area in Figure 7b indicates that a well engineered BGS is able to provide an extended linear range with even a steeper slope.

### 4.2. Experimental Implementation and Results

**Figure 8:**
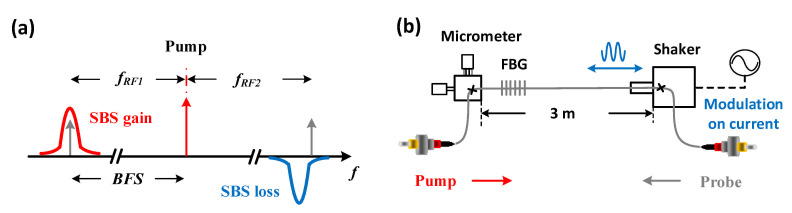
(**a**) Schematic spectrum of the multi-probe wave scheme and (**b**) specific structure of the sensing fiber.

Except that just one loss is used, the experimental implementation of the gain spectrum engineering in dynamic sensing is similar to that in static sensing. Hence, the same multi-probe wave scheme is carried out with the relative frequency between the pump and probe waves (see the schematic of Figure 8a) also following Equation (6). In order to have a comparison with the simulation results, the pump pulse width is set to 14 ns. The specific structure of the 3 m segment sensing fiber is illustrated in Figure 8b. It is anchored between a 2-dimensional micrometer stage and a shaker, which is driven by an audio signal generator and determines the longitudinal vibration amplitude and frequency. Thanks to the short fiber length, the launched power of the pump (18 dBm in average) and probe waves for gain and loss (−4.1 dBm and −2.7 dBm, respectively) can be much higher than for static measurements. An FBG with its reflection spectrum out of the pump and probe wave frequencies is inscribed on the vibrating fiber to perform an independent measurement of the vibration as a reference.

**Figure 9:**
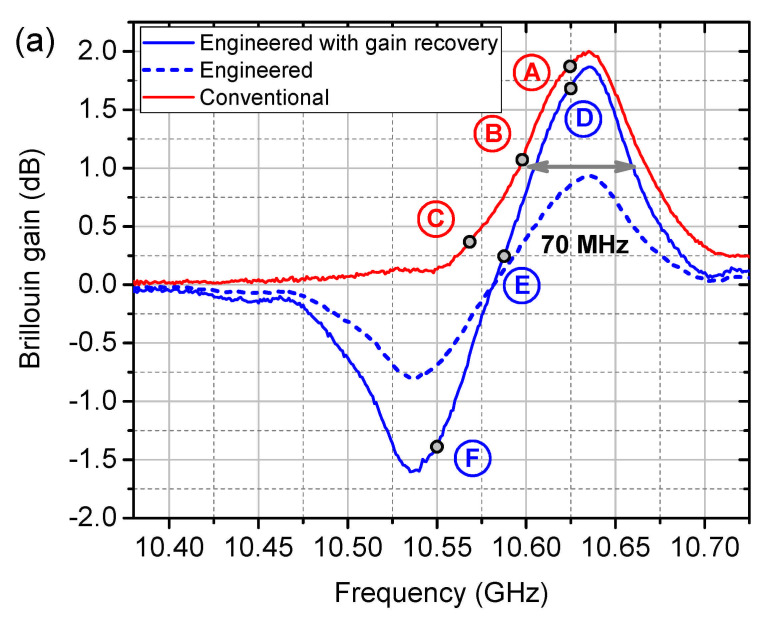

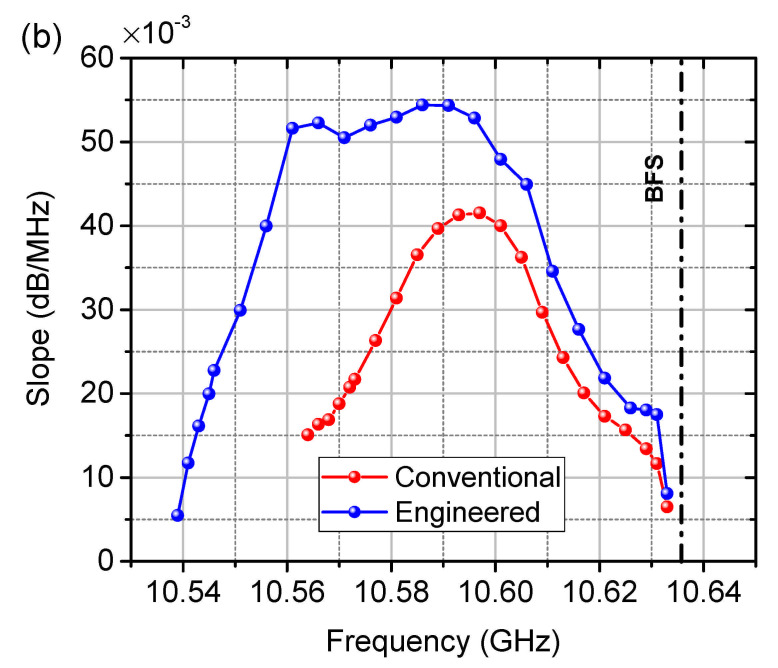
Experimental (**a**) conventional (red) and engineered (blue) BGS and (**b**) their slopes as a function of the interrogation frequency.

Before the dynamic sensing can be started, a preliminary static calibration of the setup has to be carried out [24]. Figure 9a shows the reconstructed conventional (red) and engineered (blue dashed) BGS with m=0.845 and d=1.337 as a function of the scanning RF frequency $f_{RF1}$. The gain penalty of the engineered BGS due to the dual probe wave scheme is recovered (see the discussion in Section 5) and plotted as the blue solid curve in Figure 9a. Slope-assisted dynamic measurements are carried out on three different interrogation points on each BGS (labelled from A-F in Figure 9a). Among them, A, C ($f_{RF1}$ = 10.629 GHz and 10.566 GHz, respectively) and D, F ($f_{RF1}$ = 10.626 GHz and 10.551 GHz, respectively) are at the edge of the slope, while B and E ($f_{RF1}$ = 10.597 GHz and 10.591 GHz, respectively) are in the middle of the linearity.

**Figure 10:**
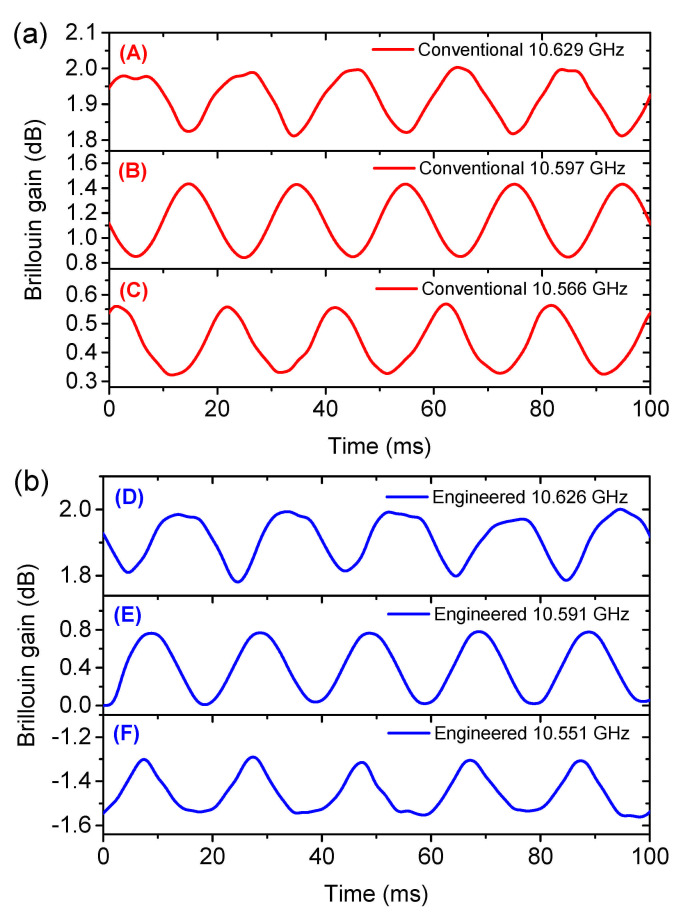
Sinusoidal Brillouin gain response of the conventional (**a**) and engineered (**b**) BGS at the interrogation frequencies in Figure 9a.

The dynamic signal to be measured is a 50 Hz longitudinal sinusoidal strain signal. As measured by the FBG, the peak-to-peak amplitude of the strain is 280 με. Figure 10 depicts the acquired sinusoidal Brillouin gain response from the conventional (red in (a)) and engineered BGS (blue in (b) with gain recovery) with 4 times of averaging. As can be clearly indicated, the sinusoidal gain signals at the middle of the linearity (interrogation point B and E) are well maintained, while severe distortions can be observed at the edges (upper edges for interrogation point A, D and lower edges for C, F). From the sinusoidal gain signal amplitude, the slope of the BGS can be calculated and plotted in Figure 9b. Please note that, the slope here is not simply the first derivative of the BGS plotted in Figure 9a. It is also showing agreement to the simulation results that the engineered BGS provides even 30% higher slope than the conventional BGS with the linear range (with a relative constant slope [28]) widely extended.

**Figure 11:**
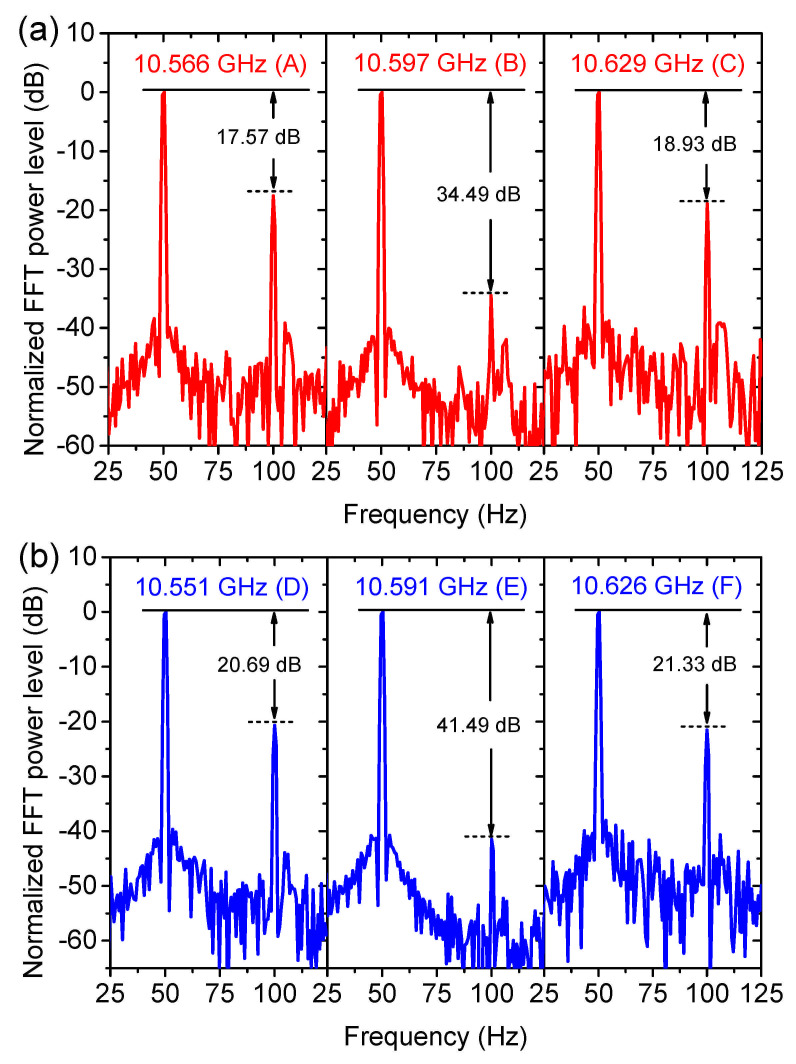
FFT spectra of the conventional (**a**) and engineered (**b**) BGS at the interrogation frequencies in Figure 9a.

In linear algebra, a function y(x) is defined as linear only if y(x1+x2)=y(x1)+y(x2) and y(ax)=ay(x). An important conclusion of these requirements is that, the output of a linear system does not include frequencies beyond those in its input. Thus, the linear range of the spectrum can be characterized by the undesired harmonic level of the Brillouin gain response after a fast Fourier transform (FFT). For the interrogation points very close to, or far away from the BFS of the conventional BGS, the second harmonic level rises (see Figure 11(a-A,a-C)). The same situation happens for the interrogation points D and F on the engineered BGS. However, the situations at the middle of the linearity are totally different. In both cases, a high suppression of the second harmonic level is achieved (34.49 dB for the conventional BGS at point B and 41.49 dB for the engineered BGS at point E).

**Figure 12:**
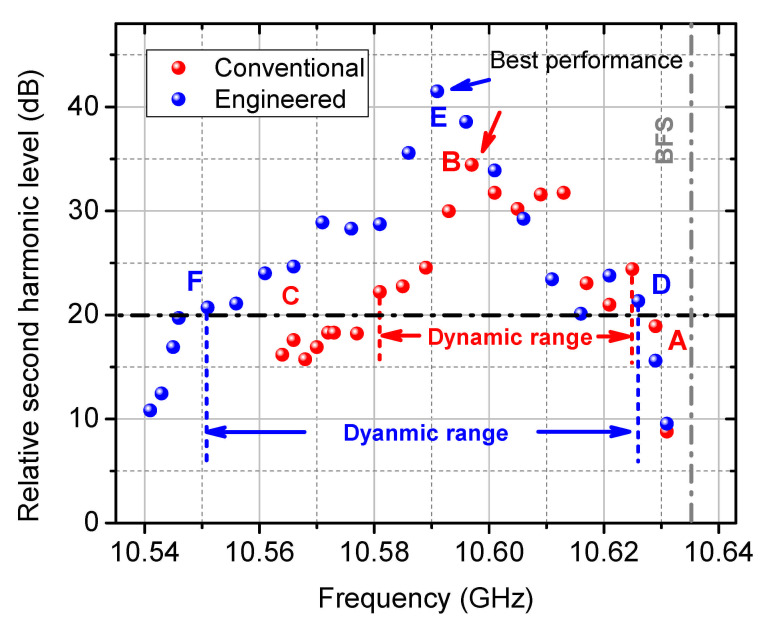
Relative second order harmonic level as a function of the interrogation frequency. A-F are the interrogation frequencies in Figure 9a.

Systematic dynamic measurements at a variety of interrogation points on both conventional and engineered BGS have been carried out. Under the same FFT analysis, the second harmonic level is plotted in Figure 12. The best performance, i.e., the ideal interrogation point of the corresponding gain spectrum, can be defined with the highest suppression of the second harmonic level. It is clearly indicated from the 7 dB higher second harmonic level at the best performance that, the engineered BGS owns a better sensing functionality than the conventional BGS. The linear dynamic range can be quantified under a specific level of tolerance of the harmonic level, e.g., 20 dB as shown by the dash-dotted horizontal line in Figure 12. Under this condition, the linear dynamic ranges are quantified to be 44 MHz and 75 MHz for the conventional and engineered BGS, respectively, i.e., an extension of the dynamic range of 70.45% has been experimentally demonstrated. It is especially important that, compared to all other range extension methods shown so far [28,29], the spectrum engineering extends the range without any compromises to the slope.

## 5. Gain Penalty and Recovery

In the experimental results of the static sensing in Section 3.3 and dynamic sensing in Section 4.2, there was a difference between the experimental Brillouin gain value acquired by the engineered BGS and the simulation. This gain penalty can be attributed to the multi-probe wave experimental implementation and can be explained by the way the Brillouin gain is calculated from the acquired BOTDA trace.

**Figure 13:**
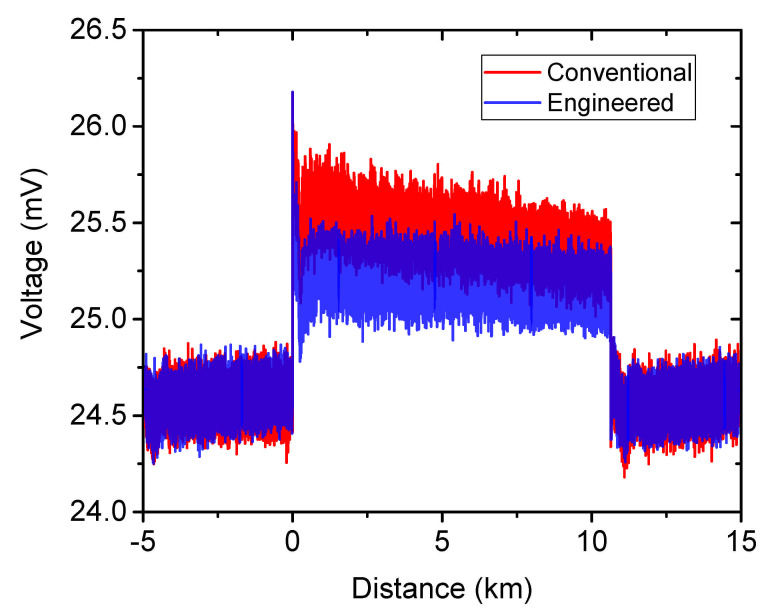
Time domain traces measured with the conventional (red) and engineered (blue) BGS at a pump-probe frequency offset of 10.605 GHz for static sensing. The voltage level of the trace with the engineered BGS is down shifted for a better visualization.

Traditionally, the BOTDA trace acquired at a fixed pump-probe frequency offset in the vicinity of BFS is composed of three parts. For static sensing in Section 3.3, for instance (the (red) trace in Figure 13), the first part (before 0 km) and the third part (after 10.7 km) of the trace (baseline), have theoretically the same voltage level. In these parts, the pump pulse is not yet interrogating the sensing fiber and hence, the voltage level represents the probe power and the Brillouin gain is around 1 in linear scale and 0 dB in logarithmic scale. For the calculation of the Brillouin gain, a division of the voltage in the second part (between 0 km and 10.7 km) with the baseline value is usually used. Since the three probe waves have the same power as the engineered BGS and are summed up in the detector with no possibility to separate, the baseline value is three times higher than the conventional BGS, leading to only 1/3 of the real engineered Brillouin gain value. To recover the correct gain with a multi-probe wave detection, the following calculation is performed.

Provided that there is no cross-interaction between the probe waves (e.g., FWM), for the simultaneous detection of multi-probe waves the logarithmic Brillouin gain is: [27]

(8) $$G_{log}=10\mathrm{lg}\left(\sum_{i=1}^{N}P_{si,SBS}/\sum_{i=1}^{N}P_{si}\right)$$

In the experiment the number of probe waves is N=3, the original probe wave power is $P_{si}$ and after the Brillouin interaction, it is changed to $P_{si,SBS}$. With an equal launching probe power, we may denote $P_{s}=P_{si}$, which leads Equation (8) to:

(9) $$G_{log}=10\mathrm{lg}\frac{\sum_{i=1}^{N}P_{si,SBS}}{N\cdot P_{s}}=-10\mathrm{lg}N+10\mathrm{lg}\left(\sum_{i=1}^{N}G_{i,lin}\right)$$

where

(10) $$G_{i,lin}=\frac{P_{si,SBS}}{P_{s}}=10^{G_{i,log}/10}\approx 1+\frac{\ln10}{10}\cdot G_{i,log}$$

is the linear Brillouin gain of the corresponding probe wave with the logarithmic Brillouin gain denoted as $G_{i,log}$, lg and ln represents the common and natural logarithm, respectively. In a BOTDA sensor the gain is always small. Thus, the exponential functions in Equation (10) can be expanded in a Taylor series with a small offset at zero gain. The substitution of Equation (10) in Equation (9) give rise to:

(11) $$\begin{array}{cc} G_{log} & =-10\mathrm{lg}N+10\mathrm{lg}\left\{N\left[1+\frac{\ln10}{10N}\cdot \left(\sum_{i=1}^{N}G_{i,log}\right)\right]\right\}\\ \; & =10\mathrm{lg}\left[1+\frac{\ln10}{10N}\cdot \left(\sum_{i=1}^{N}G_{i,log}\right)\right] \end{array}$$

Considering the Taylor series of the logarithmic function with a small offset x≪1: lg(1+x)≈x/ln10, Equation (11) can be simplified to:

(12) $$G_{log}\approx \frac{1}{N}\left(\sum_{i=1}^{N}G_{i,log}\right)$$

indicating a penalty on the logarithmic gain with a factor of *N* for a simultaneous detection with a multi-probe scheme. The equivalent linear expression of Equation (12) is given by,

(13) $$G_{lin}=10^{G_{log}/10}\approx 10^{\frac{1}{N}\cdot \frac{1}{10}\left(\sum_{i=1}^{N}G_{i,log}\right)}=10^{\frac{1}{N}\cdot \frac{1}{10}\left(\sum_{i=1}^{N}10\mathrm{lg}G_{i,lin}\right)}=\prod_{i=1}^{N}G_{i,lin}^{\frac{1}{N}}$$

The conditions for the approximations in Equations (12) and (13) are $|G_{i,log}/10|\ll$1 and $|G_{i,lin}-1|\ll$1, respectively, which are generally ensured due to the small gain of a BOTDA sensor.

While the measured gain with the engineered BGS is suffering from a penalty by a factor of N=3, according to Equation (12), the noise level is also reduced. Whether or not the SNR ultimately changes depends on whether the dominant contribution of noise comes from thermal noise during detection. The thermal noise level of a thermal noise dominant detection is independent of the input probe wave power. Therefore, the division of the signal by a factor of *N* will lead to a simultaneously decrease of the noise level also by a factor of *N*, leading to an eventually unchanged SNR and a totally equivalent sensing performance when the gain is recovered by a simple multiplication. If the noise is dominated by shot noise, however, due to its statistical behavior, the division of the signal by a factor of *N* will result in a noise level reduction only by a factor of $\sqrt{N}$, Thus, the overall SNR penalty will also be $\sqrt{N}$. When the contributions from shot and thermal noise are comparable, the influence on the noise reduction lies between a factor of $\sqrt{N}$ and *N*.

Based on the above analysis, the gain recovery of the engineered BGS by the multiplication by a factor of 3 is only justified if the following conditions are fulfilled: (a) very similar probe powers; (b) small gain approximation and (c) a thermal noise dominant detection. According to the setup configuration and the property of the BOTDA sensor, the conditions (a) and (b) are satisfied. A thermal noise dominant detection can be justified by the demonstration of the same noise level for various input powers at the detector. As shown in Figure 13, the baseline of both traces from the conventional and engineered BGS are well overlapped and show the same RMS value, indicating the same noise level though with different input powers (three times different) to the detector. Please note that, for a better comparison, the voltage level of the trace for engineered BGS (blue) is manually downshifted. Thus, the gain recovery is justified and a corresponding recovered engineered BGS is depicted in Figure 5a in gray. As demonstrated in Figure 6, the overlap of the frequency error calculated by the gain recovered engineered BGS and the one without gain recovery validates the full equivalence of the SNR.

**Figure 14:**
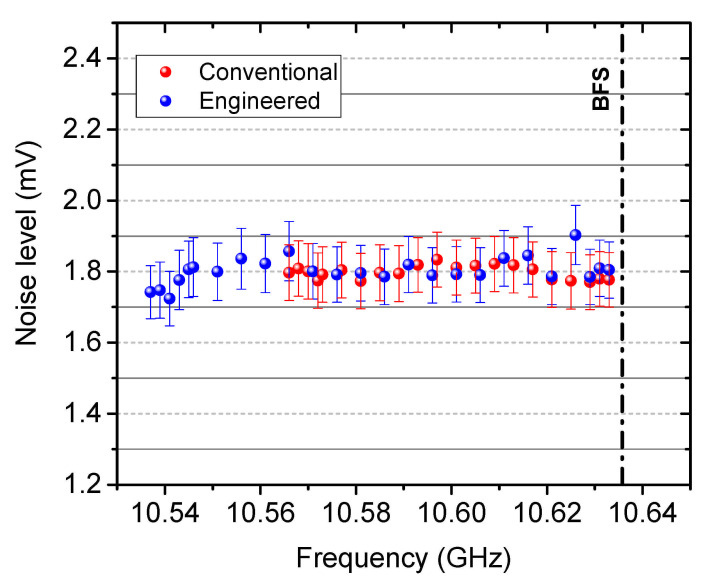
Noise level of the detected trace baseline as a function of the interrogation frequency for dynamic sensing.

The discussion above is also valid for the experimental results of dynamic sensing. A thermal noise dominant detection has to be proved for the justification of gain recovery. Therefore, the noise level at the trace baseline with the conventional BGS (single probe input before the interaction with the pump) and the engineered BGS (two probe inputs before the interaction with the pump) have been collected at different interrogation points. As demonstrated in Figure 14, without showing too much fluctuations, the mean value of the noise level with conventional and engineered BGS are 1.795 mV and 1.802 mV, respectively, showing very good agreement with the noise specification of the photodetector (1.8 mV in RMS value) within the error tolerance. Therefore, a thermal noise dominant detection and the gain recovery are justified for dynamic sensing as well.

## 6. Summary

In this paper, we have reviewed the Brillouin spectrum engineering and its application in distributed Brillouin sensing. The gain spectrum engineering has provided a novel way regarding its special spectral profile to further enhance the performance of state-of-the-art BOTDA techniques in both static and dynamic sensing tasks. For static sensing, it is for the first time to be systematically investigated that the peak detection and its robustness to the noise can be enhanced with a sharper shape of the gain spectrum and thus a higher measurand resolution can be achieved. In dynamic sensing, a solution to the major drawback, the trade-off between the dynamic range and the slope, has been proposed with an engineered gain spectrum, leading to a simultaneous enhancement of both maximum and minimum detectable measurand of a slope-assisted sensor. Table 1 shows a summary of the benefits that can be provided to static and dynamic Brillouin sensing if spectrum engineering is used. These achievements are of great importance to and might drastically benefit a variety of demanding sensing tasks in structural health monitoring, pipeline surveillance and homeland security.

## Figures and Tables

**Table 1:** Summary of the key benefits of spectrum engineering in BOTDA and SA-BOTDA in comparison to standard systems.

**Static BOTDA**	
Sensing range extension	40.4%
Measurand resolution enhancement	2 times
Noise level	The same
**Dynamic SA-BOTDA**	
Dynamic range extension	70.45%
Slope enhancement	30%
Noise level	The same

## Data Availability

Not applicable.
